# Interconnected hydrologic extreme drivers and impacts depicted by remote sensing data assimilation

**DOI:** 10.1038/s41598-023-30484-4

**Published:** 2023-02-28

**Authors:** Timothy M. Lahmers, Sujay V. Kumar, Kim A. Locke, Shugong Wang, Augusto Getirana, Melissa L. Wrzesien, Pang-Wei Liu, Shahryar Khalique Ahmad

**Affiliations:** 1 Hydrological Sciences Lab, NASA Goddard Space Flight Center (NASA-GSFC), Greenbelt, MD USA; 2 Earth System Science Interdisciplinary Center (ESSIC), University of Maryland, College Park, MD USA; 3 Science Applications International Corporation (SAIC), McLean, VA USA; 4 Science Systems and Applications Inc. (SSAI), Lanham, MD USA

**Keywords:** Hydrology, Natural hazards

## Abstract

Hydrologic extremes often involve a complex interplay of several processes. For example, flood events can have a cascade of impacts, such as saturated soils and suppressed vegetation growth. Accurate representation of such interconnected processes while accounting for associated triggering factors and subsequent impacts of flood events is difficult to achieve with conceptual hydrological models alone. In this study, we use the 2019 flood in the Northern Mississippi and Missouri Basins, which caused a series of hydrologic disturbances, as an example of such a flood event. This event began with above-average precipitation combined with anomalously high snowmelt in spring 2019. This series of anomalies resulted in above normal soil moisture that prevented crops from being planted over much of the corn belt region. In the present study, we demonstrate that incorporating remote sensing information within a hydrologic modeling system adds substantial value in representing the processes that lead to the 2019 flood event and the resulting agricultural disturbances. This remote sensing data infusion improves the accuracy of soil moisture and snowmelt estimates by up to 16% and 24%, respectively, and it also improves the representation of vegetation anomalies relative to the reference crop fraction anomalies.

## Introduction

In a changing climate, the likelihood of hydrologic extremes has been increasing^1–3^, as climate change can impact both means and extremes^4^ of hydrologic cycle processes, potentially resulting in an increased frequency of floods in some regions^5,6^ and decreases in others^7^. In a warming world, the physical processes that affect hydrologic response, such as rain-on snow runoff events, are also changing, such that the seasonality of streamflow has been shifting^8,9^. The geography of rain-on-snow runoff events is predicted to move from low to high elevations^10,11^. In addition to floods, there is also potential for an increase in dry extremes in a warming world with increased drought frequency and occurrences in many parts of the world^12–14^. The increased frequency of drought and heatwave events is expected to have consequences such as escalating crop failures in future projection scenarios^1^. Thus, the consensus of literature shows that climate change is increasing the magnitude and frequency of extreme hydrologic events, and the human influence in many of these events is substantial.

Understanding the drivers and impacts of extreme events in a warming climate requires models and observations of complex physical processes. Often, conceptual models alone are insufficient to resolve the heterogeneity and the associated complexity of land surface processes. The significant human footprint and related impacts further compound the difficulty of accurate characterization of the terrestrial water cycle. When available, observations, particularly from remote sensing, offer independent and spatially distributed information on such processes, which can be used to constrain modeled estimates. This infusion of remote sensing data into a model is typically achieved through data assimilation (DA) efforts that blend observational information with modeled estimates by accounting for their relative errors and uncertainties.

There have been numerous efforts to incorporate observations of surface datasets of soil moisture^15,16^, snow^17^, land surface temperature^18^, and vegetation^19–21^ in land surface models (LSMs). These studies demonstrate that surface DA can significantly improve the representation of related LSM states and fluxes. Several studies have also examined the utility of remote sensing DA for hydrologic modeling. For example, bias corrected leaf area index (LAI) and evapotranspiration (ET) within the variable infiltration capacity (VIC) model improved the characterization of streamflow estimates for a case study in the Connecticut River Basin^22^. Recent work has demonstrated added value of Gravity Recovery And Climate Experiment (GRACE) terrestrial water storage (TWS) for improving streamflow simulations in West Africa^23^. The benefit of assimilating snow products has been widely demonstrated for both improving modelled snow water equivalent^24^ (SWE) and for hydrologic response^17,24–29^. Many of these studies also documented the potential utility of DA of remote sensing measurements for characterizing hydrological extremes^30,31^.

These efforts on land surface DA suggest that use of satellite remote sensing-based constraints in LSMs adds substantial value over models alone^32,33^. Note that much of the literature outlined above focuses on the assimilation of univariate datasets, whereas similar multivariate and multi-sensor DA applications are less common. There is a benefit of joint assimilation of soil moisture and vegetation data for realizing moisture and evaporative fluxes on the land surface^20,34^, as well as for drought monitoring^35^. The assimilation of LAI^36^ and snow cover fraction^37^ combined with streamflow can also improve hydrologic model states compared to univariate DA. Similarly, assimilation of LAI and surface soil moisture improves model estimates over that from a control simulation^38^. The assimilation of both soil moisture and snow datasets resulted in improvements to other model process variables and for drought estimation^39^. Furthermore, joint assimilation of (Soil Moisture and Ocean Salinity) SMOS soil moisture and GRACE TWS has been shown to produce more consistent improvements in water cycle flux estimation^16^.

These prior studies are focused on quantifying the aggregate impact of DA (with one or two datasets at most), whereas the examination of the utility of DA for specific extreme events is lacking to the best of our knowledge. Such quantifications are also important to highlight the utility of remote sensing information, and thus this work is novel because we consider assimilation of three remote sensing datasets during an extreme event that resulted in substantial financial losses. Extreme events such as floods and droughts often also have large economic impacts. Therefore, large scale and timely information can be significantly more valuable in protecting property and the populace. Here we consider such an event, the 2019 Mississippi River Basin flood, to demonstrate how DA of multivariate and multi-sensor remote sensing datasets help in capturing the drivers and impacts of the flood.

**Figure 1:**
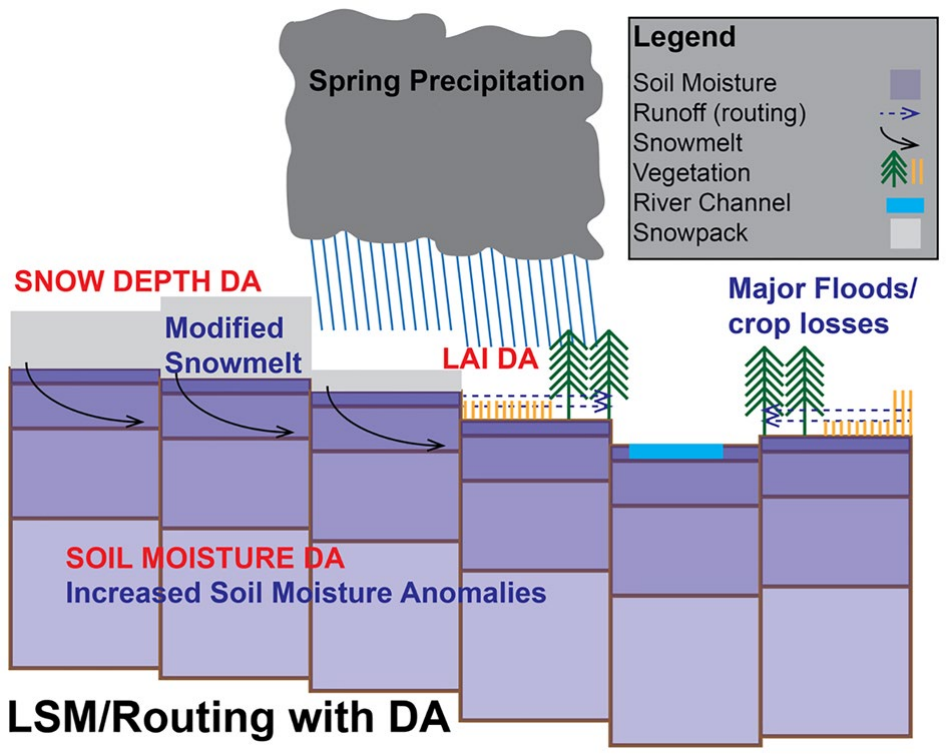
Conceptual illustration of the LSM and Routing Model with the impacts of DA. Physical processes are labeled in blue, while the impacts of DA are labeled in red. This graphic was generated by the authors using Adobe Illustrator.

Extreme events such as the 2019 Mississippi River Basin Flooding follow a complex triggering process, which often includes a progression of anomalies in meteorological and hydrologic processes such as snowmelt, antecedent soil moisture, and rainfall. This complexity also extends to impacts on soil moisture, evaporation, runoff, and vegetation, which can be challenging to estimate given the limitations of models^40^. We demonstrate that both advanced earth observing systems, in addition to LSMs and hydrologic models, are needed to adequately capture the progression and consequences of such extreme events. Our LSM system is forced with remote sensing based precipitation from the (Integrated Multi-satellitE Retrievals for GPM) IMERG system. Furthermore, we use soil moisture retrievals from NASA’s Soil Moisture Active Passive (SMAP) mission^41^, LAI from the NASA Moderate Resolution Imaging Spectroradiometer (MODIS) aboard the Terra and Aqua missions, and snow depth retrievals from the Advanced Microwave Scanning Radiometer 2 (AMSR-2) mission for resolving the 2019 Mississippi basin flood event and its impacts. We consider the effects of the individual DA of each dataset on the model, as well as multivariate DA by including all three datasets simultaneously. The present study makes use of the NASA Land Information System^42,43^ (LIS) with the Noah-MP v4.0.1 LSM^44^ coupled to the Hydrological Modeling and Analysis Platform (HyMAP) surface routing model^45^. Figure 1 shows a schematic of how various DA components influence the modification of snow melt, soil moisture, and vegetation states. In this figure, the model configuration is illustrated, and the impacts of specific DA instances are noted in larger red text. Physical processes that were significant for this event are also noted in larger blue text.

In this article, we demonstrate how an LSM with DA of remote sensing information is able to resolve the drivers and impacts of this event. The use of remote sensing data as both forcing and through data infusion to resolve an extreme event makes this study novel. This article is organized as follows. Section "Observed progression of 2019 Mississippi basin flood event" outlines the progression of the 2019 Mississippi Basin flooding through observation datasets. In Section "Results and analysis", we demonstrate the results of our modeling study, including the necessity for remote sensing constraints to resolve the full progression of the 2019 floods and impacts of the DA on the hydrology. Section "Discussion and conclusion" includes a discussion of the implications of these results for the hydrologic and remote sensing communities, including possible future work. Section "Methods" describes the Open-Loop and DA LIS simulations, including the Ensemble Kalman Filter (EnKF) DA settings.

## Observed progression of 2019 Mississippi basin flood event

**Figure 2:**
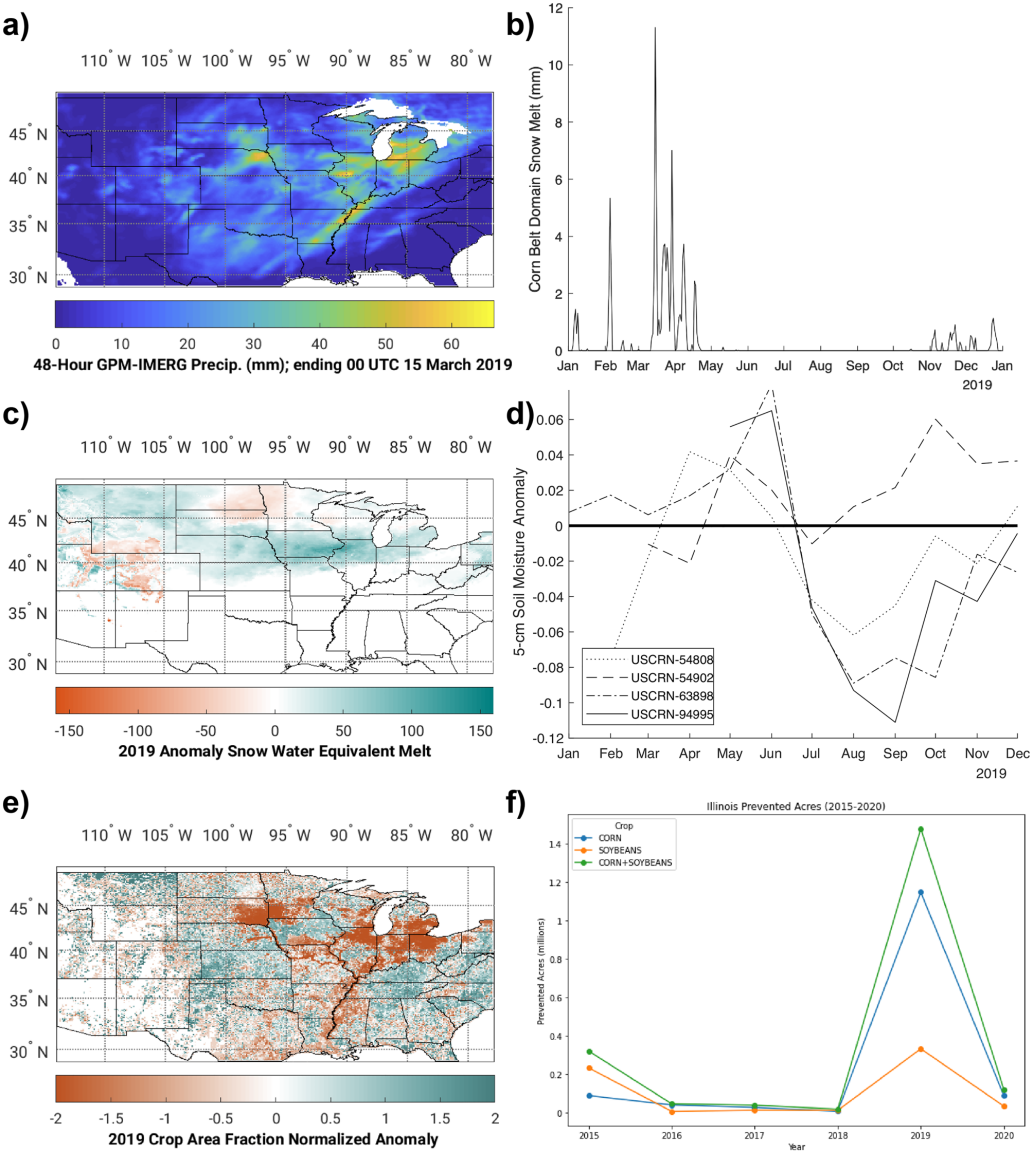
Observed Impacts of the 2019 Mississippi Basin Flood Event. IMERG Precipitation for March 14–15 (top left, **a**), University of Arizona SWE melt over the corn belt domain (top right, **b**), University of Arizona (Dawson et al. 2016) SWE snowmelt anomalies (compared to 2016–2020) for 10 March to 29 March 2019 (middle left, **c**), USCRN soil moisture anomalies for four sites in the corn belt domain (middle right, **d**), 2019 Crop Area Fraction for Soy and Corn Standardized Anomalies based on the CropScape dataset (bottom left, **e**), and USDA Farm Service Agency (FSA) prevented acres (bottom right, **f**) from 2015 through 2020 for the State of Illinois are shown. Map panels were generated by Matlab version r2017b.

Similar to other recent extreme events, the Mississippi Basin Flood event was preceded by both meteorological and hydrologic anomalies (Fig. 2). The first major disturbance was an anomalous rainfall event that affected much of the region in mid-March 2019. Rainfall estimates during the event, derived from the NASA IMERG dataset, are shown in Fig. 2a (March 14–15). Note that the IMERG realization of this event is consistent with the NLDAS2 precipitation estimates^46^, shown in Supplementary Fig. S1. NLDAS2 is considered a high quality product over CONUS given that it also includes radar and gage derived precipitation^46^.

**Figure 3:**
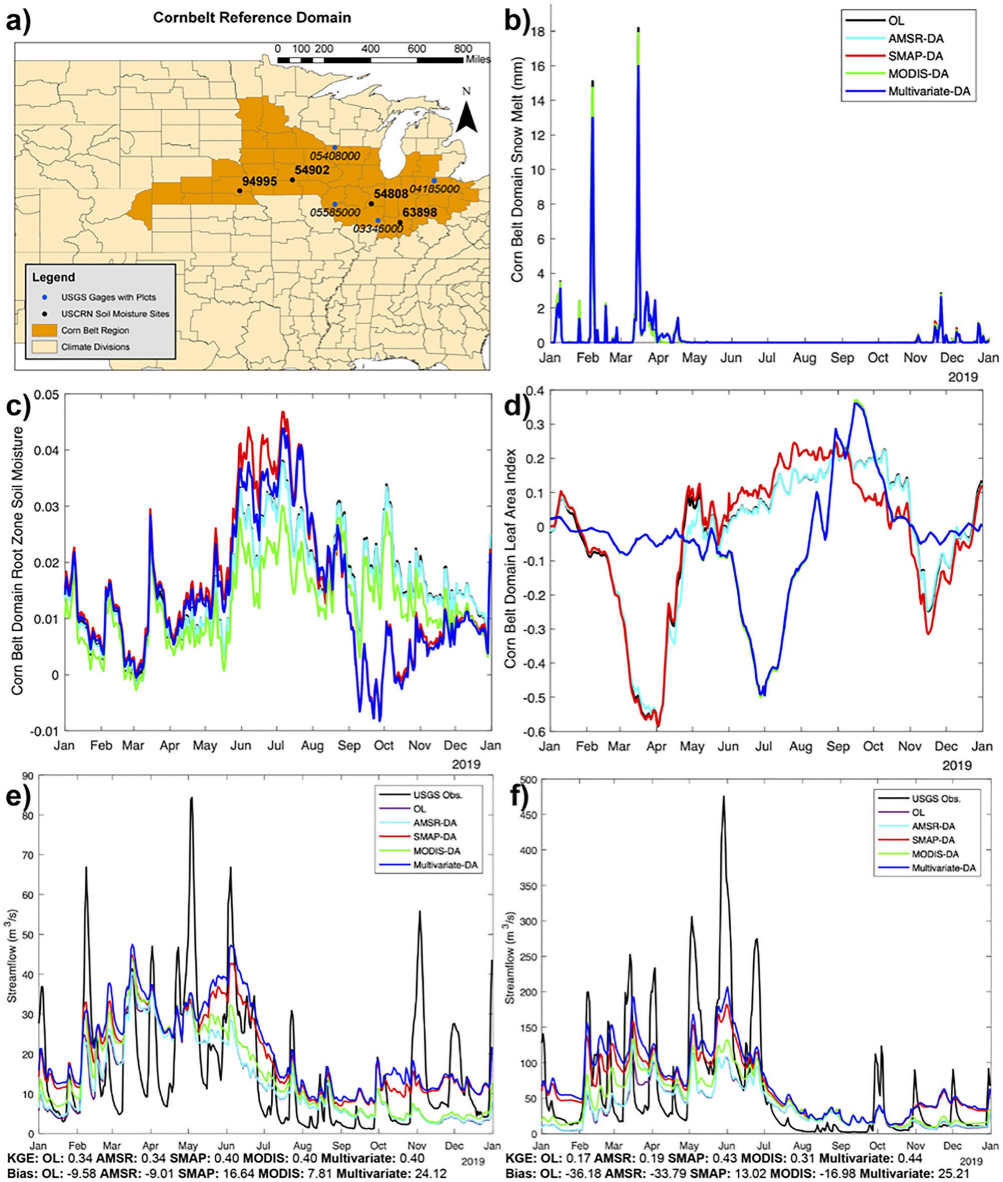
Timeseries of modeled anomalies over the Corn Belt Region. The Corn Belt domain with USCRN gages (black w/bold text) and plotted USGS gages (blue w/italic text) (**a**), snow melt (**b**), soil moisture anomalies (**c**), and LAI anomalies (**d**) timeseries are shown. Panels (**b**) through (**d**) include the open-loop (OL), AMSR-DA, SMAP-DA, MODIS-DA, and multivariate DA timeseries. Sample hydrographs for 2019 with USGS observations, OL, AMSR-DA, SMAP-DA, MODIS-DA, and multivariate DA are shown for USGS gages 04185000 in Northern Ohio (**e**) and 05585000 in Illinois (**f**). The GIS panel was generated using ArcMap v.8.1.

This extreme rainfall resulted in a rain-on-snow event with high values of snowmelt, from the abnormally high snow amounts during the 2018–2019 winter across the basin. Snowmelt, which we represent here as change in SWE, over the Corn Belt region (denoted by NOAA Climate Regions associated with corn crops; highlighted in Fig. 3a) from the University of Arizona Snow Dataset^47^ is also shown in Fig. 2b. Prior studies have documented that the University of Arizona Snow Dataset has high skill compared to several reference products^48^. For example, it had a 0.98 correlation and 30% mean absolute deviation compared with the ASO dataset^49^. The University of Arizona product was also found to have a 0.78 correlation and 31% mean absolute deviation with airborne gamma SWE aircraft measurements over the North-Central US^50^. Note that we assume all negative changes in SWE from the University of Arizona dataset are melt, rather than other processes such as compaction or sublimation, as the University of Arizona dataset does not include a melt product. The spatial distribution of snowmelt anomalies (also shown as loss of SWE) from March 10 to 29, 2019, compared to the years 2016–2020 (snowmelt or loss of SWE is positive) from the University of Arizona Snow Dataset is shown in Fig. 2c. In Fig. 2c., the March snowmelt is primarily concentrated over a domain from Eastern Nebraska through Northwest Indiana, consistent with the Corn Belt Region (Fig. 3a.) and much of the March rainfall (Fig. 2a). Note that the Corn Belt domain is based on NOAA Climate Regions in the Central US that are designated by the National Center for Environmental Information (NCEI) as part of the Corn Belt (available online at: https://www.ncdc.noaa.gov/monitoring-references/maps/us-ag-belts.php#corn).

The snowmelt from the rain-on-snow combined with above average spring precipitation had a significant contribution to the elevated soil moisture. Monthly soil moisture anomalies (compared to the full period from 2016 to 2020) from four US Climate Reference Network (USCRN) stations with complete data over the affected region (Fig. 3a.) are shown in Fig. 2d, and they demonstrate the above average soil moisture for the region during Spring 2019. These observations show wetter than average soil moisture during the typical planting and early growing seasons, with some drying in late summer and into the fall season. Note that most of these gages are located further south and west within the Corn Belt domain, where snowmelt (and likely increased soil moisture) was less substantial.

This above average soil moisture prevented the planting of corn and soy crops throughout the Corn Belt region and resulted in major crop losses and lower yields compared to prior years (Fig. 2e,f). This contributed to total losses from the whole flood event of over $20 Billion^51^. Figure 2e shows the spatial impacts of these losses. Here, 2019 crop fraction of both corn and soy, derived from USDA Cropland Data Layer (CDL) dataset, is plotted as a standardized anomaly (i.e. [crop fraction – mean] / standard deviation) compared to crop years 2016–2020. With the exception of much of Iowa, this panel shows that crop losses largely followed the snowmelt and extreme precipitation over the corn belt region. Figure 2f shows prevented acres of corn and soy for the state of Illinois, the core of the Corn Belt region affected by the 2019 flooding. This figure shows relative increases of prevented acres (i.e., areas where crops were not grown), especially for corn crops but also to a lesser extent for soy.

Thus, the impacts and the progression of the 2019 flooding (starting from precipitation and snowmelt anomalies to saturated soils and finally crop losses) are clearly shown in satellite and in-situ observations. In the next section, we demonstrate how LSM simulations resolve this event with the infusion of information from remote sensing.

## Results and analysis

As demonstrated in Fig. 2 in the previous section, the 2019 Mississippi Basin flooding started with an extreme precipitation event. The extreme precipitation combined with rapid snowmelt increased soil moisture to levels that resulted in delays in—and some cases preventing—the planting of crops, causing crop losses throughout the Corn Belt. Figure 3 shows time series of snowmelt, soil moisture anomalies, and LAI anomalies (compared to 2016–2020 simulations) from all of the LIS simulations (OL, AMSR-DA, SMAP-DA, MODIS-DA, and multivariate-DA), averaged over the Corn Belt region (Fig. 3a). Figure 3 demonstrates how these processes and anomalies progressed throughout 2019 in five different LSM simulations, including the OL simulation initialized on 1-January 1980, and with DA starting from the OL simulation on 1-April 2015. DA combinations include AMSR-2 snow depth, SMAP soil moisture, MODIS LAI, and all three products as a multivariate DA simulation.

Consistent with observed UA snowmelt (Fig. 2b), Fig. 3b shows that nearly all the simulations (regardless of DA) depict the spring snowmelt in March 2019. Table 1 shows skill scores for the time series of spatially averaged daily simulated SWE-melt compared to computed UA SWE melt, and there are statistically significantly improved correlation coefficients (from 0.726 to 0.810) and reduced root mean square error (RMSE) by 24% in the DA solutions (AMSR-2 and Multivariate) that include snow. This is despite slightly higher percent bias of SWE melt, thus showing some added value of AMSR-2 snow DA.

**Table 1:** Model Skill Compared to Observations. Skill score metrics (bias, correlation coefficient, and RMSE) for modeled 2019 LIS OL, AMSR-DA, SMAP-DA, MODIS-DA, and Multivariate-DA simulated SWE melt over the Corn Belt domain compared to UA SWE melt are plotted in the top rows. July 2015–July 2020 0–10 cm soil moisture from the same LIS simulations compared to 5-cm soil moisture at USCRN sites are listed in the bottom rows. 95% confidence intervals for all metrics are shown. Confidence intervals for bias and RMSE use a student’s t-test and a Chi-Square Distribution, respectively. Confidence intervals for correlation are computed using a Fisher transform.

Skill metric	LIS OL	LIS AMSR-DA	LIS SMAP-DA	LIS MODIS-DA	LIS MV-DA
2019 SWE melt					
Bias (mm/day)	0.019 (− 0.086 to 0.125)	0.023 (− 0.056 to 0.102)	0.018 (− 0.086 to 0.122)	0.017 (− 0.087 to 0.120)	0.024 (− 0.056 to 0.103)
Correlation	0.726 (0.674 to 0.771)	0.811 (0.773–0.844)*	0.723 (0.670–0.769)	0.724 (0.671–0.770)	0.810 (0.772–0.843)*
RMSE	1.021 (0.952–1.101)	0.767 (0.715–0.827)*	1.010 (0.942–1.089)	1.005 (0.937–1.084)	0.772 (0.720–0.832)*
USCRN 54902					
Bias (soil volumetric water content)	− 0.019 (− 0.021 to − 0.017)	− 0.019 (− 0.021 to − 0.017)	− 0.010 (− 0.012 to − 0.008)*	− 0.006 (− 0.008 to − 0.004)*	− 0.003 (− 0.005 to − 0.001)*
Correlation	0.814 (0.797–0.830)	0.815 (0.798–0.831)	0.812 (0.795–0.828)	0.795 (0.776–0.812)	0.797 (0.779–0.815)
RMSE	0.043 (0.041–0.044)	0.043 (0.041–0.044)	0.041 (0.040–0.042)*	0.041 (0.040–0.043)*	0.041 (0.040–0.042)*
USCRN 54808					
Bias (soil volumetric water content)	− 0.019 (− 0.023 to − 0.016)	− 0.020 (− 0.023 to − 0.016)	− 0.010 (− 0.013 to − 0.007)*	− 0.022 (− 0.025 to − 0.019)	− 0.019 (− 0.022 to − 0.016)
Correlation	0.788 (0.769–0.805)	0.789 (0.770–0.806)	0.782 (0.763–0.800)	0.706 (0.681–0.729)*	0.705 (0.680–0.728)*
RMSE	0.067 (0.065–0.070)	0.067 (0.065–0.070)	0.067 (0.065–0.070)	0.071 (0.069–0.074)	0.071 (0.068–0.073)
USCRN 63898					
Bias (soil volumetric water content)	− 0.028 (− 0.030 to− 0.025)	− 0.028 (− 0.030 to − 0.025)	− 0.024 (− 0.026 to − 0.022)	− 0.026 (− 0.028 to − 0.023)	− 0.025 (− 0.027 to − 0.023)
Correlation	0.824 (0.808–0.838)	0.823 (0.807–0.837)	0.832 (0.818–0.846)	0.815 (0.799–0.830)	0.816 (0.800–0.831)
RMSE	0.060 (0.058–0.062)	0.060 (0.058–0.062)	0.058 (0.056–0.060)	0.056 (0.054–0.058)	0.056 (0.054–0.058)
USCRN 94995					
Bias (soil volumetric water content)	− 0.067 (− 0.070 to − 0.063)	− 0.066 (− 0.070 to − 0.063)	− 0.052 (− 0.056 to − 0.049)*	− 0.054 (− 0.057 to − 0.051)*	− 0.052 (− 0.055 to − 0.049)*
Correlation	0.585 (0.550–0.618)	0.587 (0.552–0.620)	0.605 (0.571–0.637)	0.716 (0.689–0.740)*	0.717 (0.691–0.741)*
RMSE	0.093 (0.090–0.097)	0.093 (0.090–0.097)	0.083 (0.080–0.086)*	0.079 (0.076–0.082)*	0.078 (0.075–0.081)*

The magnitude of the soil moisture anomalies is greater in the SMAP soil moisture DA and the multivariate DA cases by late spring (Fig. 3c). The DA simulation also begins to capture drying conditions later in the summer, with the progression of summer soil moisture anomalies consistent with station observations shown in Fig. 2d. This shows that the OL simulation (and other simulations without soil moisture DA) tend to underestimate the soil moisture anomalies from the 2019 flood event. The SMAP soil moisture DA solution shows the most pronounced positive soil moisture anomalies, while the positive soil moisture anomalies in the multivariate solution are greater than the OL simulation but lower than the SMAP DA simulation. Table 1 shows skill scores for Noah-MP surface soil moisture (i.e., 0–10 cm soil depth) compared to 5-cm soil moisture measurements from the same four USCRN sites in Fig. 2d for 2016 through 2020. These results show reduced bias and RMSE at site numbers 54902, 63898 and 94995 (statistically significant at sites 54902 and 94995). These reductions in bias and RMSE are as high as 21% and 16%, respectively, at site number 94995. Statistically significant improvements to correlation also occur at site 94995. DA slightly degrades model performance at site number 54808 (correlation is significantly reduced).

As saturated soils resulted in crop losses, LAI anomalies, a proxy for changes in green vegetation, are shown in Fig. 3d. In this panel, it is clear that the simulations without LAI DA fail to show decreased LAI over the Corn Belt as LAI anomalies are positive. For the LAI and multivariate DA simulations, negative anomalies associated with crop losses are present over the Corn Belt domain. The negative LAI anomaly for 2019 (compared to the years 2016–2020) in the LAI DA and multivariate DA simulation shows that there is reduced vegetation in the simulations with LAI DA, following the 2019 floods. This suggests that the model with LAI DA is able to capture the crop losses, which were a result of human activity (not represented by Noah-MP without data assimilation), as were shown previously from USDA data (Fig. 2). Note that other DA instances (i.e. SWE and soil moisture) have little impact on the LAI timeseries, likely due to the role of dynamic vegetation in Noah-MP, which is not as sensitive to the other modes of DA.

**Figure 4:**
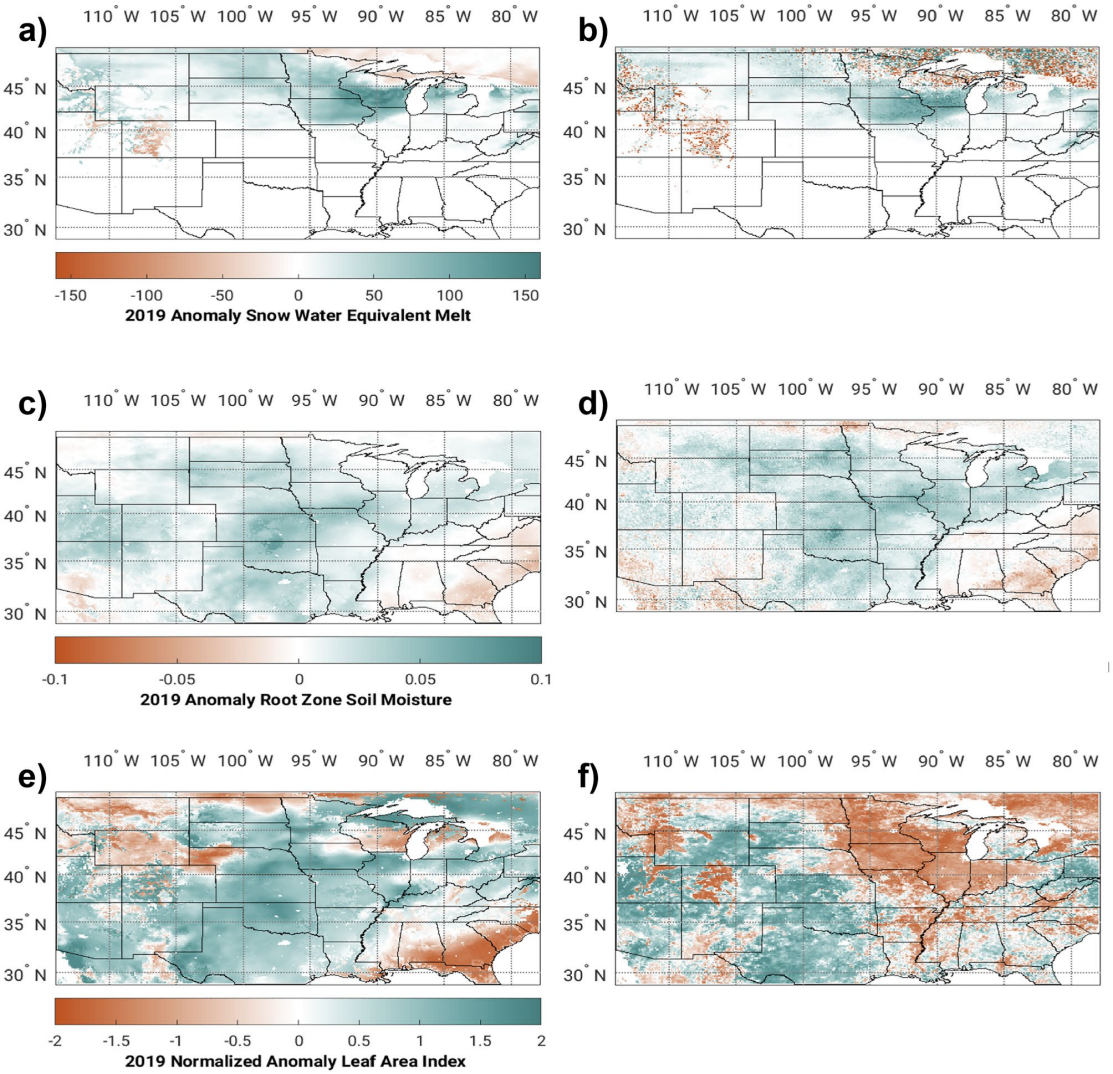
Spatial Extent of Modeled 2019 Mississippi Basin anomalies compared to averages from 2016 to 2020. Snow melt anomalies (10 March–29 March) from the LIS-OL simulation (**a**), snow melt anomalies (10 March–29 March) from the LIS-DA simulation (**b**), soil moisture anomalies (1 May–1 July) from the LIS-OL simulation (**c**), soil moisture anomalies (1 May–1 July) from the LIS-DA simulation (**d**), LAI standardized anomalies (1 June–1 July) from the LIS-OL simulation (**e**), and LAI standardized anomalies (1 June–1 July) from the LIS-DA simulation (**f**) are plotted. The color bars for the OL simulations (left) are the same as for the DA simulations (right). Map panels were generated by Matlab version r2017b.

The effects of DA are also realized in the spatial anomalies (compared to 5-year averages from 2016 to 2020 when all datasets were available) of surface variables (Fig. 4). For SWE melt and soil moisture, anomalies are compared to the average and not normalized (i.e., WY2019—Average), while LAI anomalies are normalized by standard deviation (i.e., standardized anomalies). Absolute model values are also shown in supplementary Material (Fig. S2). In Fig. 4a,b, snowmelt anomalies from March 10 to 29 are shown for the OL (left) and multivariate DA (right) simulations. Both simulations show a significant area of anomalously high snowmelt near the Corn Belt area; however, the OL solution has more snowmelt than is depicted in the University of Arizona observations (shown in Fig. 2c). The multivariate DA simulation has reduced high bias, and the snowmelt extends further west towards Nebraska, as in the observations. We also compared spatial similarity between modeled and observed SWE melt using the structural similarity index (SSIM)^52^. SSIM between UA observations and the LIS-OL simulation is 0.42, which increases to 0.44 with multivariate DA. Note that a gaussian smoother with a sigma value of 2 was applied to the data to remove discontinuities in the data fields before computing SSIM.

Soil moisture anomalies for May and June 2019 are shown in Fig. 4c,d, and this Figure shows (as in Fig. 3) that the multivariate DA simulation has more pronounced soil moisture anomalies over the Corn Belt compared to the OL solution. The added value of DA is best realized by comparing the standardized LAI anomalies in Fig. 4e,f to the change in crop fraction in Fig. 2e, as the losses of corn and soil crops shown in Fig. 2e mostly follow the negative LAI anomalies in the multivariate solution in Fig. 4f. The SSIM between the LAI standardized anomalies compared to observed crop fraction standardized anomalies (Fig. 2e) is 0.06 in the OL simulation and is increased to 0.07 with multivariate DA. Rather than depicting reduced LAI from crop losses, the OL solution shows positive LAI anomalies (Fig. 4e), thus demonstrating the added value of LAI DA. This inconsistency between the OL and DA simulations reflects the lack of human processes (i.e., delayed or prevented planting) in Noah-MP.

This progression of extremes over the Corn Belt, examined both temporally (Fig. 3) and spatially (Fig. 4) demonstrates how the cascade of hydrologic anomalies associated with the 2019 floods, which were realized by observations in Fig. 2, are depicted in the LSM with multivariate DA. These results demonstrate that the model is unable to resolve these processes without DA of relevant surface variables. Therefore, these results show the utility of remote sensing products combined with the LSM model for depicting the cascade of hydrologic extremes during this extreme flood event.

The impacts of the different DA variables and the full multivariate DA on streamflow are considered in Supplementary Fig. S3. Figure S3 shows the Kling-Gupta-Efficiency^53^ (KGE) and percent bias skill scores of the multivariate DA LIS-HyMAP streamflow compared to available USGS gages-II observations within the Corn Belt domain. The analysis period for these metrics is 2015–2020. Note that only gages with limited human modifications (i.e., reference basins) are considered. These results show that LIS-HyMAP has high skill scores across the south and east parts of the Corn Belt; however, KGE drops below zero (indicating lower skill) further west. In several areas, this is associated with both increased high and low biases. The middle and bottom panels show changes in skill score (also KGE and bias) of the multivariate DA and soil moisture DA, respectively, compared to the OL simulation. The changes in model skill are similar in both cases, and they demonstrate that multivariate DA improves model skill, and most of this change is from soil moisture DA (see bottom panels of Supplementary Fig. S1). Multivariate DA also increases model streamflow bias (in many cases this is negative bias going to near zero), such that DA increases model streamflow (i.e., the OL simulations have negative bias that is reduced from DA). Sample hydrographs (gages shown in Fig. 3a) from 2019 in two basins are plotted in Fig. 3e,f, and an additional two basins are plotted in Supplementary Fig. S4. USGS gage 04185000 (Tiffin River at Stryker, OH; Fig. 3e) and USGS gage 05585000 (La Moine River at Ripley, IL; Fig. 3f) have higher drainage areas and associated with improved KGE following DA. For both of these basins, KGE is improved with multi-variate DA, and negative bias is reduced. Therefore, the impacts of flooding in 2019 are more substantial in the DA simulation. Note that some positive bias is added with multiple instance of DA. USGS gage 03346000 (North Fork Embarras River near Oblong, IL; Supplementary Fig. S4) and USGS gage 05408000 (Kickapoo River at La Farge, WI; Supplementary Fig. S4) have lower drainage areas (and less skill overall), but show similar patterns of improved KGE following DA.

All of these solutions show a systematic tendency for LIS-HyMAP to produce too much baseflow, while limiting surface runoff events. The choice of the TOPMODEL runoff scheme in Noah-MP (see details of LSM settings in section "Methods") likely limits the skill of the streamflow simulations^54,55^. This tendency is particularly noticeable in higher order basins. These results are also consistent with the mixed KGE skill shown in Supplementary Fig. S3.

## Discussion and conclusion

This analysis demonstrates the added value of multivariate DA from remote sensing datasets on the simulation of a hydrological extreme event; the 2019 Midwest floods. The improvements to model performance are demonstrated by the reduction of soil moisture RMSE of up to 16% and snowmelt RMSE of up to 24% (Table 1) and by the increases to structural similarity for model snow melt and LAI, compared to observed snow melt and crop fraction anomalies.

The impacts on physical processes enabled by DA (illustrated in Fig. 1) demonstrate that the DA solution can capture increased soil moisture anomalies and resultant flooding and crop losses. Comparison of the OL simulations with the DA simulations suggests the OL simulation fails to capture the precise location of snowmelt anomalies over the Midwest (Fig. 3), as well as the translation of snow and precipitation anomalies to soil moisture and crop losses. The latter two biases (i.e., SWE and soil moisture) likely reflect systematic biases of the Noah-MP LSM snow and soil moisture schemes; however, the lack of crop losses in the OL simulation are due to agriculture impacts not being parameterized by Noah-MP. More broadly, this suggests that the modelled relationships from extreme precipitation and snowmelt to soil moisture and the effect of saturated soil on crop growth are not sufficiently resolved by the Noah-MP LSM. Given these differences between the OL and DA results, this analysis demonstrates the benefits of remote sensing DA for use within modeling environments that are used to inform management decisions around extreme events.

Given the limitations of the OL compared to the multivariate DA solution, these results demonstrate potential benefits of future high resolution remote sensing measurements. The added value of different surface datasets used in the DA framework corroborate prior work showing improvements to streamflow from snow and LAI DA^22,27,29,56^. Previous work has also demonstrated added value of LAI and soil moisture DA for resolving ET anomalies over the continent of Australia^34,38^. Thus, the current study adds to the body of literature demonstrating the benefits of remote sensing infrastructure for resolving the processes leading up to natural disasters, including floods and droughts. This is potentially beneficial for improving physical process understanding through models and for monitoring extreme events for operational stakeholders. Future work could extend the elements of the present study by incorporating surface DA with LSMs to better understand physical processes associated with hydrologic extremes such as floods^30,31^, fires^57^, and droughts^13^. Streamflow DA could also help to resolve the impacts of diversions and other human modifications to hydrologic systems. Assimilation of data from the recently launched NASA Surface Water and Ocean Topography (SWOT) mission could help to address this issue in the future. Future work could also include varying LSMs to explore the limits of specific models and adjusting LSM parameters and parameterizations to better capture hydrologic response during extreme events.

## Methods

The NASA LIS system was used in the present study, as it supports DA^58,59^, multiple LSMs, and streamflow routing through the HyMAP routing model^45^. The LIS LSM and DA system is well suited to resolve this and other extreme events, as it has been successfully demonstrated in several DA scenarios, specifically in the assimilation of surface soil moisture^39,58,59^, snow^24–26,39^, leaf area index^20,57^, and other surface variables^18,57,60^.

Version 4.0.1 of the Noah-MP LSM^44^ is used with dynamic vegetation, and the runoff scheme is TOPMODEL with groundwater^44^. Noah-MP is run at 10-km grid resolution for the full Mississippi River basin domain (28.55°–49.95° N, 77.95°–113.95° W). Noah-MP uses four soil levels with depths (from top to bottom) of 0.1, 0.3, 0.6 and 1.0 m. The OL simulation was spun up from 1 January 1980 and executed through 1 January 2021 with MERRA2 atmospheric forcing and IMERG precipitation (when available starting in 2002). HyMAP streamflow routing is added to the OL simulation starting 1 January 2000 and continued through 1 January 2021. As the SMAP dataset does not begin until early 2015, the AMSR-2 DA, SMAP DA, and full multi-variate DA simulations were all started on 1 April 2015 with initial conditions from the OL simulation. The MODIS-DA simulation was initialized from the OL simulation on 1 July 2002, at the start of the MODIS LAI dataset.

LIS uses a 1-dimensional Ensemble Kalman Filter^61^ (EnKF) for DA. This DA method is used for all three datasets and the multivariate DA. EnKF has been widely used in prior studies^61–66^, and it enables the DA to account for both model errors and non-linear tendencies within the model processes. EnKF additionally allows for the characterization of model errors with an ensemble, and it is capable of resolving non-linear model dynamics and discontinuities in temporal observations^61^. EnKF DA has also been demonstrated to add value to model variables in a theoretical case study, where forcing and model biases were introduced, with snow and soil moisture^65^. The EnKF process alternates between the model forecast timestep and the analysis update step. EnKF is executed starting with the forecast timestep (i.e., running the LSM forward). In Eq. 1, let the forecast timestep be (k) and *ƒ*su the prior analysis timestep be (k−1). Subscripts (i) and (j) are the x and y grid points in the model domain, respectively. When the LSM is run, the model state (for any given variable) at a grid point is projected forward from (${\widehat{\mathbf{x}}}_{\mathbf{k}-1,\mathbf{i},\mathbf{j}}^{+}$) to the LSM state at k (${\widehat{\mathbf{x}}}_{\mathbf{k},\mathbf{i},\mathbf{j}}^{-}$). The analysis step is then computed as follows:

$${\widehat{{\varvec{x}}}}_{{\varvec{k}},{\varvec{i}},{\varvec{j}}}^{+}={\widehat{{\varvec{x}}}}_{{\varvec{k}},{\varvec{i}},{\varvec{j}}}^{-}+{{\varvec{K}}}_{{\varvec{k}},{\varvec{i}},{\varvec{j}}}\left({\widehat{{\varvec{y}}}}_{{\varvec{k}},{\varvec{i}},{\varvec{j}}}-{{\varvec{H}}}_{{\varvec{k}},{\varvec{i}},{\varvec{j}}}{\widehat{{\varvec{x}}}}_{{\varvec{k}}}^{-}\right)$$

Here, the posterior state (${\widehat{\mathbf{x}}}_{\mathbf{k},\mathbf{i},\mathbf{j}}^{+}$) combines the observation state (${\widehat{{\varvec{y}}}}_{{\varvec{k}},{\varvec{i}},{\varvec{j}}}$) and the a priori state (${\widehat{\mathbf{x}}}_{\mathbf{k},\mathbf{i},\mathbf{j}}^{-}$). In this equation, ${{\varvec{H}}}_{{\varvec{k}},{\varvec{i}},{\varvec{j}}}$ represents the observation operator that relates the model states to the observation. ${{\varvec{K}}}_{{\varvec{k}},{\varvec{i}},{\varvec{j}}}$ is the Kalman gain, which weights the impact of forecast innovations $\left({\widehat{{\varvec{y}}}}_{{\varvec{k}},{\varvec{i}},{\varvec{j}}}-{{\varvec{H}}}_{{\varvec{k}},{\varvec{i}},{\varvec{j}}}{\widehat{{\varvec{x}}}}_{{\varvec{k}},{\varvec{i}},{\varvec{j}}}^{-}\right)$ in the analysis update. Kalman gain is based on the model and observation error covariances.

The DA methods and settings used here follow previous work^57^, and the settings for forcing perturbations and model variable perturbations are shown in Supplementary Table S1. The forcing perturbations are the same for the snow depth, soil moisture, LAI, and multivariate DA simulations, and these settings follow prior applications of EnKF DA^59,67^. Note that the state vectors for snow depth, soil moisture, and LAI, are all independent and executed as separate instances of EnKF DA. All DA cases have 20 ensemble members. The 20-member ensemble configuration is sufficient to avoid sampling errors for snow DA, and has been demonstrated in prior work^26^. The forcing state perturbation is 1-h, and the model state perturbation is 3-h. Observation state perturbations depend on the data temporal resolution and are 3-h for soil moisture and snow depth, and 24-h for LAI. Perturbations to atmospheric forcing account for cross-correlation in space and time between variables, which can better simulate the effects of model uncertainty^25^.

For snow depth DA, AMSR-2 microwave snow depth retrievals are used, and this is constrained by MODIS snow cover. The Snow Depth and SWE variables of Noah-MP were updated for the AMSR-2 assimilation step. Both of these variables are also perturbed in the DA step (see Table S1 in Supplementary Material). Previous work demonstrated that this method could improve simulated snow states across North America^24^. Furthermore, the Noah-MP snow scheme adds value over the earlier Noah LSM, as it uses a 3-layer snow model that accounts for the full energy balance of the snow pack^44^. This generally improves the performance of the Noah-MP LSM for resolving snowpack^68–72^.

SMAP soil moisture^20,41,57^ is used for soil moisture assimilation. SMAP soil moisture is downscaled to 1-km using the Thermal Hydraulic of Soil Moisture Disaggregation (THySM) algorithm^73^, which is also available online through the USDA Crop-CASMA system (https://nassgeo.csiss.gmu.edu/CropCASMA/). When available, both ascending (6 PM) and descending (6 AM) paths of data are used for assimilation. As THySM SMAP product is based on surface soil moisture, the shallowest layer (0–10 cm) of Noah-MP is updated. The LIS “anomaly correction” algorithm is used to correct any biases in the SMAP product^74^.

The MCD15A2H Version 6 MODIS product^75^ is used for LAI assimilation. While the MODIS MCD15A2H Version 6 product has an 8-day temporal resolution, a smoothing algorithm within the LIS system is used, so that assimilation may be performed daily.

For all the model experiments, means and standard deviations for the model variables (including soil moisture and LAI) are computed from 2016 through 2020. While we acknowledge this is a short analysis period, it is necessary because of the limits of the DA datasets (SMAP is only available from April 2015 to present). For analysis, SWE melt, soil moisture, and LAI are considered in 2019. SWE melt and soil moisture analysis use anomalies (analysis year—mean), and LAI analysis uses standardized anomalies (analysis year–mean)/standard deviation, respectively.

SSIM for SWE melt and LAI is computed using the SSIM function of the python scikit package (available online at: https://scikit-image.org/docs/stable/auto_examples/transform/plot_ssim.html), and gaussian smoothing was performed using the ndimage.filters.gaussian_filter function of the python SciPy package (available online at: https://docs.scipy.org/doc/scipy-0.14.0/reference/generated/scipy.ndimage.filters.gaussian_filter.html). Note that this additional step was necessary since EnKF does not include smoothing when DA is performed, and smoothing of model and observation data eliminates the influence local-scale discontinuities and errors in the DA and observation based datasets.

## Supplementary Information

Supplementary Information.

## Data Availability

The NASA LIS Model is available online at: https://github.com/NASA-LIS/LISF. The MERRA and IMERG atmospheric forcing and precipitation datasets are located online at https://disc.gsfc.nasa.gov/datasets?project=MERRA-2 and https://gpm.nasa.gov/data/directory respectively. USGS streamflow data are stored at: https://waterservices.usgs.gov/ and University of Arizona SWE data are available at: https://nsidc.org/data/nsidc-0719/versions/1. USCRN soil moisture data are available online at https://www.drought.gov/data-maps-tools/us-climate-reference-network-uscrn-soil-temperature-and-soil-moisture, and USDA CropScape data are available at: https://nassgeodata.gmu.edu/CropScape/. The MODIS LAI product is available at: https://lpdaac.usgs.gov/products/mcd15a2hv006/. AMSR-2 snow depth data are available at: https://nsidc.org/data/au_si12/versions/1, and SMAP soil moisture are available at: https://nsidc.org/data/smap. The datasets used and/or analyzed during the current study available from the corresponding author on reasonable request.
