# Supplementary material for: Interconnected hydrologic extreme drivers and impacts depicted by remote sensing data assimilation
Source: Sci Rep. 2023 Feb 28;13:3411. doi: 10.1038/s41598-023-30484-4 (PMC9975208; doi:10.1038/s41598-023-30484-4)
Supplement: Supplementary file 1 — Supplementary Information. [file 41598_2023_30484_MOESM1_ESM.pdf]

Interconnected hydrologic extreme drivers and impacts depicted by remote sensing data assimilation

***Timothy M. Lahmers[1,2], Sujay V. Kumar[1], Kim A. Locke[1,2] Shugong Wang[1,3], Augusto Getirana[1,3], Melissa L. Wrzesien[1,2], Pang-Wei Liu[1,4], Shahryar Khalique Ahmad[1,3]**

[1]Hydrological Sciences Lab, NASA Goddard Space Flight Center (NASA-GSFC), Greenbelt, MD, USA.

[2]Earth System Science Interdisciplinary Center (ESSIC), University of Maryland, College Park, MD, USA.

[3]Science Applications International Corporation (SAIC), McLean, VA, USA.

[4] Science Systems and Applications Inc. (SSAI), Lanham, MD, USA.

*Corresponding Author Email: timothy.lahmers@nasa.gov

Supplementary Information

Table S1 includes the perturbation settings for each variable used for data assimilation (DA), including AMSR-2 snow depth, SMAP soil moisture, and MODIS LAI. All of these settings are used for multivariate DA, and the DA settings are discussed in detail in the methods section of the manuscript.

The 48-hour rainfall plotted in Figure 2 (based on GPM-IMERG data) is also plotted from the NLDAS2 datasets in Supplementary Material (Figure S1). A different color scale is used in these figures, due to an extreme event over Nebraska in the NLDAS2 solution. Note that the NLDAS2 dataset includes gage and radar data (e.g. Xia et al., 2012). Figure S1 shows that the GPM-IMERG data follows the same spatial patterns and magnitudes as the NDLAS2 product over the most affected states (i.e. Iowa through Indiana and further North into Wisconsin). The NLDAS2 product does show a heavier rainfall event further west into Nebraska; however, this is outside of the area most affected by subsequent crop losses (as shown in later manuscript figures).

Figure S2 depicts LIS-OL and LIS-DA snowmelt, soil moisture, and LAI model values without any normalization, as in Figure 4. These results demonstrate how the model handled the progression of the 2019 extreme event.

Figure S3 includes skill scores for the LIS-HyMAP simulations with multivariate DA (top), and the impact of multivariate DA on model skill compared to the open-loop (OL) control simulation is shown in the middle panels of Figure S3. The change in skill score for only the soil moisture DA on streamflow is shown in the bottom panels. These results demonstrate how remote sensing DA (mostly due to soil moisture) impacted model skill through the Kling Gupta Efficiency (KGE) and water balance, through changes to bias.

Additional sample hydrographs from LIS-HyMAP simulations with different DA settings (compared to USGS streamflow observations) are shown in Figure S4. All of these results are discussed in detail in the Results and Analysis section of the manuscript.

References:

Xia, Y., et al. (2012), Continental-scale water and energy flux analysis and validation for the North American Land Data Assimilation System project phase 2 (NLDAS-2): 1. Intercomparison and application of model products, *J. Geophys. Res.*, 117, D03109, doi:10.1029/2011JD016048.

Table S1: Parameters for meteorological forcing and model state variables for EnKF configuration.

Variable	Perturbation Type	Std. Dev.	Cross Correlation across variables			
**Meteorological Forcing**			**SW corr**	**LW corr**	**PCP corr**	
Downward Shortwave (SW)	Multiplicative	0.3	1.0	-0.5	-0.8	
Downward Longwave (LW)	Additive	30	-0.5	1.0	0.5	
Precipitation (PCP)	Multiplicative	0.5	-0.8	0.5	1.0	
**Noah LSM snow states**			**SWE**	**snod**		
SWE	Multiplicative	0.01	1.0	0.9		
Snow depth (snod)	Multiplicative	0.01	0.9	1.0		
**Noah LSM soil moisture states**			**Layer 1**	**Layer 2**	**Layer 3**	**Layer 4**
Soil Moisture Layer 1	Additive	0.1	1.0	0.0	0.0	0.0
Soil Moisture Layer 2	Additive	0.1	0.0	1.0	0.0	0.0
Soil Moisture Layer 3	Additive	0.1	0.0	0.0	1.0	0.0
Soil Moisture Layer 4	Additive	0.1	0.0	0.0	0.0	1.0
**Noah LSM LAI states**			**LAI**			
Leaf Area Index (LAI)	Additive	0.01	1.0			

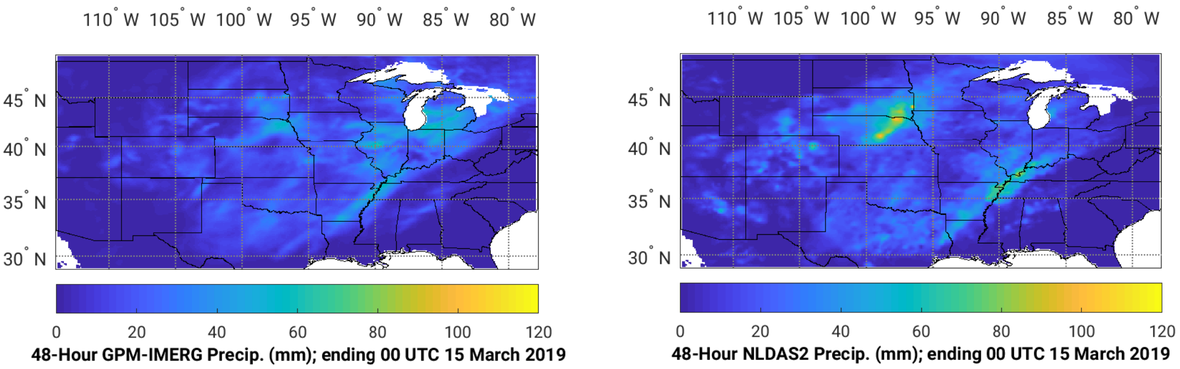

Figure S1: GPM IMERG Precipitation (left) and NLDAS2 Precipitation for the March 14 - 15 time period (right). Map panels were generated by Matlab version r2017b.

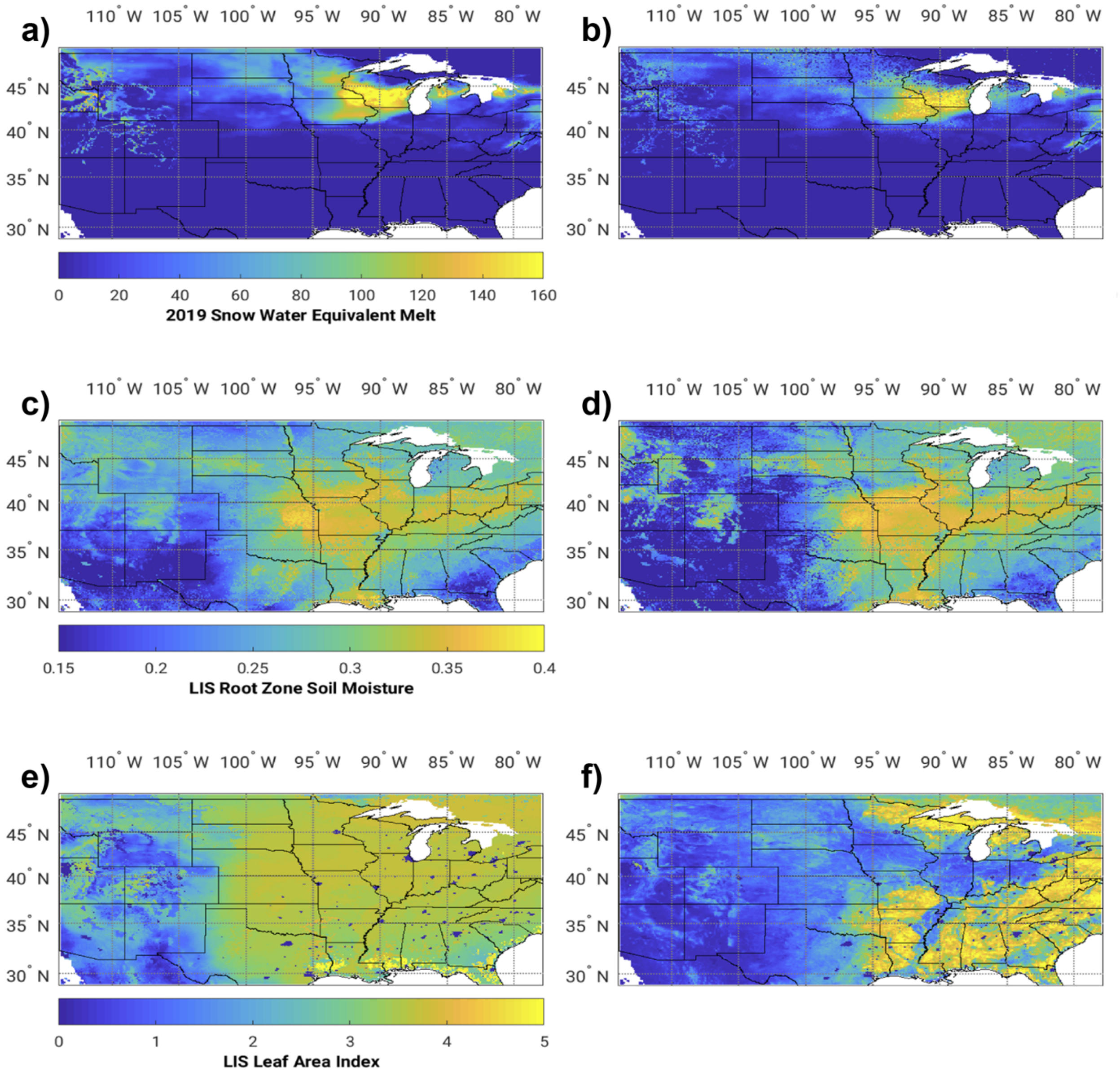

Figure S2: Modeled 2019 Mississippi Basin quantities. Snow melt (10 March – 29 March) from the LIS-OL simulation (a), snow melt (10 March – 29 March) from the LIS-DA simulation (b), soil moisture (1 May – 1 July) from the LIS-OL simulation (c), soil moisture (1 May – 1 July) from the LIS-DA simulation (d), LAI (1 June – 1 July) from the LIS-OL simulation (e), and LAI (1 June – 1 July) from the LIS-DA simulation (f) are plotted. Map panels were generated by Matlab version r2017b.

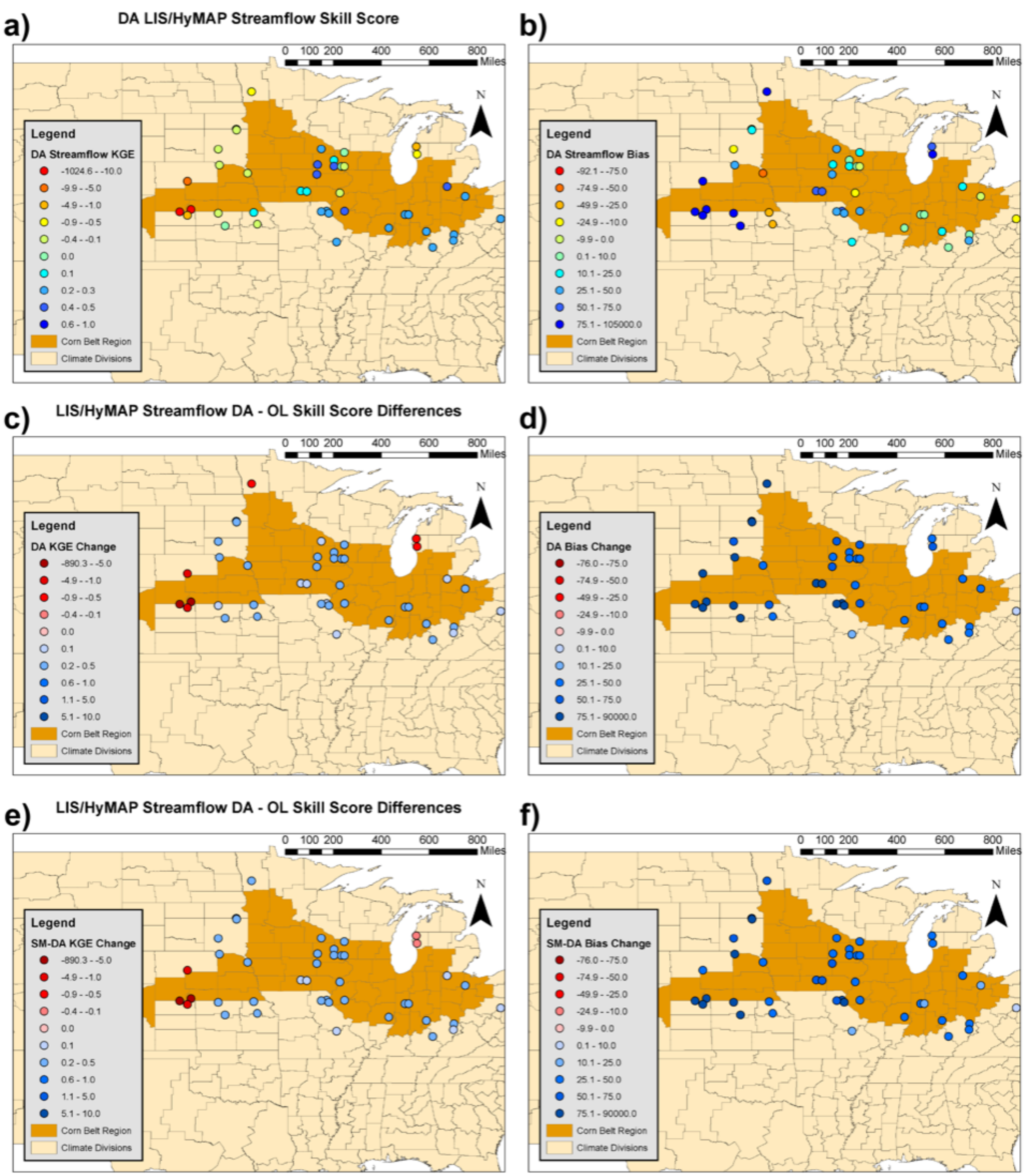

Figure S3: LIS-HyMAP multivariate-DA KGE (a), multivariate-DA Bias (b), multivariate-DA – OL KGE (c), multivariate-DA – OL Bias (d), soil moisture-DA – OL KGE (e), and soil moisture-DA – OL Bias (f), are shown for USGS References Gages in and near the Corn Belt domain. GIS panels were generated using ArcMap v.8.1.

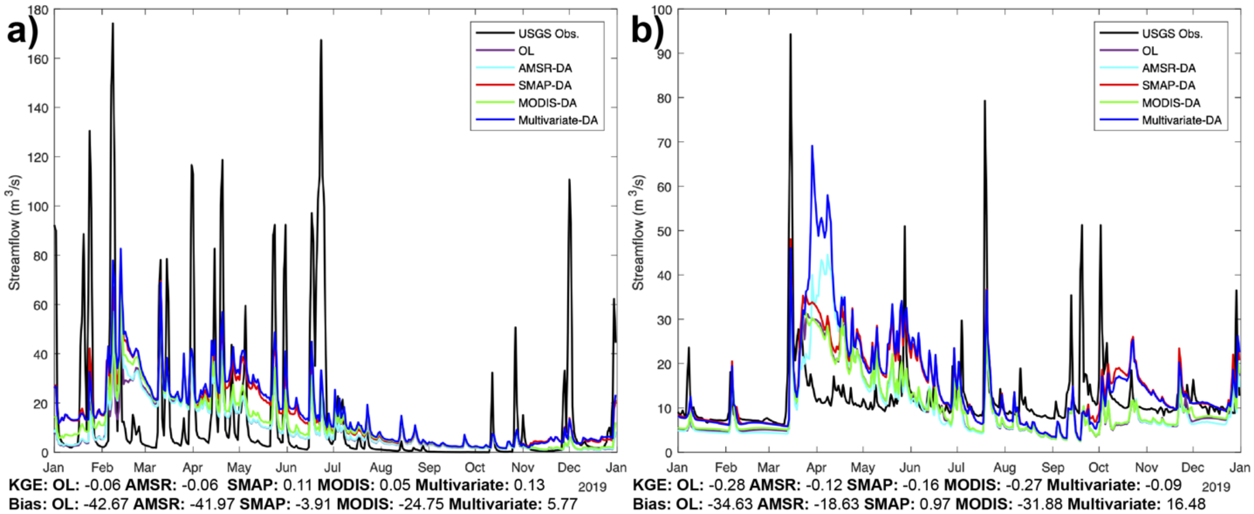

Figure S4: Additional Sample hydrographs for 2019 with USGS observations, OL, AMSR-DA, SMAP-DA, MODIS-DA, and multivariate DA are shown for USGS 03346000 in Indiana (left) and USGS 05408000 in Wisconsin (right). Map panels were generated by Matlab version r2017b.
